# Lakes Drying and Their Adverse Effects on Human Health: A Systematic Review

**Published:** 2019-02

**Authors:** Homayoun SADEGHI-BAZARGANI, Hamid ALLAHVERDIPOUR, Mohammad ASGHARI JAFARABADI, Saber AZAMI-AGHDASH

**Affiliations:** 1. Road Traffic Injury Research Center, Tabriz University of Medical Sciences, Tabriz, Iran; 2. Clinical Psychiatry Research Center, Department of Health Education & Promotion, Tabriz University of Medical Sciences, Tabriz, Iran; 3. Research Center of Psychiatry and Behavioral Sciences, Department of Health Education & Promotion, Tabriz University of Medical Sciences, Tabriz, Iran; 4. Tabriz Health Services Management Research Center, Health Management and Safety Promotion Research Institute, Tabriz University of Medical Sciences, Tabriz, Iran; 5. Iranian Center of Excellence in Health Management, School of Management and Medical Informatics, Tabriz University of Medical Sciences, Tabriz, Iran

**Keywords:** Effects, Drying, Lakes, Human health, Cohort studies, Iran

## Abstract

**Background:**

One of the most important effects of many drying lakes in the world is increasing the emergence and outbreak of different diseases. For this sake, the present study aimed to systematically review the effects of lakes drying on human health.

**Methods:**

The present systematic review was designed and conducted in 2017. Data were gathered by searching the Science Direct, Cochrane Library, Google Scholar, Scopus, PubMed, and Web of Knowledge databases, along with hand search of key journals and unpublished resources and contact with experts. There was no specific time span for the search.

**Results:**

Overall, 22 articles were selected with 20 articles about Aral Lake drying. Almost all studies were cross-sectional and retrospective. In 8 studies, the participants were children. Seventeen articles lakes drying have adverse effects on human health. Based on the type of effect, the studies were classified into 7 categories (respiratory problems, reproductive system problems, kidney and urological diseases, cancers, anemia, and diarrhea).

**Conclusion:**

Most studies depicted the harmful effects of lakes draught on human health; they had low level of evidence as they were mostly retrospective and cross-sectional. There is not enough evidence to accept or reject with high level of certainty the very effects of lakes drying on human health. To provide such evidence we suggest conducting middle and long term cohort and observational studies with scientific bases.

## Introduction

Humans have made great efforts in identification and control of adverse effects of natural disasters in recent years and achieved partial success in this regard (1,2). Owing to these successes, today, the adverse effects of earthquakes, floods, volcanoes, and other disasters have become less frequent when compared with the past. One notable success was the prevention and decrease in the level of adverse effects of such phenomena on human health (3–5). Among different types of disasters lakes drying and more specifically that of salt lakes is a case to which fewer interventions happened and we have trivial related information about it (6).

Of the most important effects of lakes, drying is the emergence and increasing prevalence of diseases that brings unwanted experiences to humans (7, 8). Prevalence of diseases in neighboring regions of Aral Lake is the epitome of the discussed subject matter. Studying the global experiences like Aral Lake shows that farmers used herbicide, insecticides, and chemical fertilizers to fertilize their lands, and on the other hand agricultural sewage has entered the lake and its sedimentation made the produced particles and dust to be polluted. Of prevalent diseases are tuberculosis, respiratory diseases, asthma, eye diseases, pharynx and larynx diseases, kidney and liver diseases, cancers, typhoid, hepatitis, brucellosis, diseases caused by deficiencies in minerals and vitamins, diarrhea, infectious diseases, giving birth to deformed infants, arthritis, impaired endocrine function, neurological and behavioral changes, immune system diseases, mental retardation, and delayed puberty increased by lakes drying (9–15). Raised dusts from the basin of Lake Owens in California, the US, contained elements like sodium sulfate, sulfur, arsenic, chrome, cobalt, nickel, lead and etc. and caused allergy and respiratory diseases, asthma, sinus infection, headache, ear infection, bronchitis, eye pain, sore throat, coughing, fatigue, lung cancer and cardiovascular diseases (16,17).

Our earth has experienced drying of many of its lakes. Among them are Lake Urmia in Iran, Aral Lake in central Asia region, Salton Lake in California, Lake Owens in California, and Great Salt Lake in Utah State; the US. Yet, there is no clear cut and strong evidence about the effect of lakes drying on human health. Therefore, establishing well-designed studies with high level of credibility in their methodology would be helpful. For this reason, we need to review the existing literature and experiences of other countries.

Thus, the present study was conducted to provide a systematic review of what has been investigated about the effects of lakes drying on human health.

## Methods

This systematic review was conducted in 2017 according to review approach is taken from the book titled “Systematic Reviews to Support Evidence-Based medicine” (18).

### Search strategy

Data were gathered by searching the Cochrane Library, Google Scholar, Scopus, PubMed, Web of Knowledge, and Science Direct databases up to December 2017. To identify and cover more articles, hand search of some credible key journals also performed. After excluding irrelevant articles, the references list of the included articles searched to increase the credibility. To search the grey literature we looked at European Association for Grey Literature Exploitation (EAGLE) and Health Care Management Consortium (HMIC).

### Inclusion and exclusion criteria

Inclusion criterion was: those papers that somehow pointed to lake drying and its effects on health. The papers or other sources that discussed drying of other kinds of water supplies including rivers, lagoons, and ponds were excluded from the study. Moreover, articles in languages other than Persian and English were excluded (except for some papers with English abstracts having useful data). Moreover, did not include those articles dealing with only how to face the effects of lakes drying and no possible correlation with health was assessed. Moreover, those articles not resulting from research projects (merely a personal expression of subject matter or still a hypothesis) were excluded.

### Quality assessment

Two assessors independently assessed the reporting quality of articles by STROBE (Strengthening the Reporting of Studies in Epidemiology Observational) checklist. This checklist was used because it was merely used to evaluate observational studies and was credible and its Persian translation was available (19). The checklist acquired 22 items in abstract, introduction, method, results and discussion sections (20, 21). Controversies between assessors were referred to a third researcher. Articles that did not report more than 50% of items in the checklist were excluded.

### Data extraction

To extract data we firstly designed data extraction form in Microsoft Word. Data from 3 articles were extracted as pilot and then the form revised. The extracted data included: first author, year of publication, country, name of the lake, study design, sample size, participants, health problem and summary of main findings.

### Data Analysis

Both data analysis and reporting were done manually. Content- Analysis was used for categorizing the results. It is a method for analysis of qualitative information extracted from articles. The process of content-analysis included: reaching information, creating initial themes, categorizing the themes, obtaining consensus on the accuracy of categorization.

## Results

**Fig. 1:**
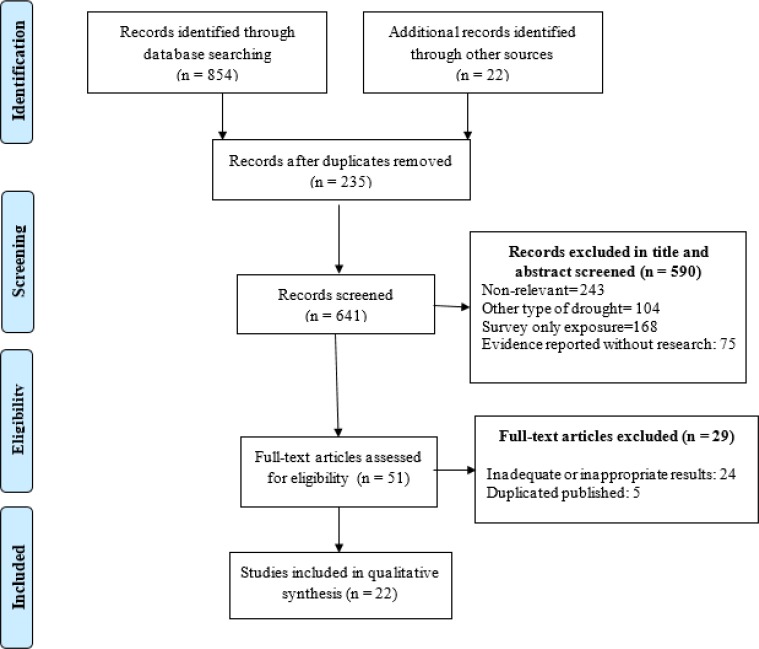
Searches and inclusion process

From 876 records we excluded 235 due to duplication. We also excluded 590 abstracts and titles and 29 full texts and finally selected 22 articles (Fig. 1).

Characteristics of the included studies are summarized in Table 1. The studies were done in Uzbekistan (11 studies), Kazakhstan (9 studies), The United States (1 study), and Canada (1 study). Twenty studies were about Aral Lake, and 2 others were for Great Salt Lake and Old Wives Lake, respectively in The United States and Canada. Almost all studies were cross-sectional and case-control. Children compromised the participants of 8 studies.

**Table 1:** Characteristics of articles that studied the effects of lakes drying on human health

**Author, Year**	**Country**	**Lake**	**Study design**	**Sample size & Participants**	**Health problems**	**Conclusion**
Hashizume M, et al: 2004 (22)	Kazakhstan	Aral	Cross-sectional	97 students	Anemia	Iron absorption problems causing anemia
Hashizume M, et al: 2003 (23 )	Kazakhstan	Aral	Cross-sectional	468 students	Anemia	Iron deficiency was one main reason for anemia in the region
Mamyrbayev A, et al: 2016 (13 )	Kazakhstan	Aral	case-control	All kinds of childhood cancers from 2004 to 2011	Childhood Cancers	The rate of childhood cancers in Aral lake region was higher than control group but not different with national statistics
Bilyalova Z, et al: 2012 (24 )	Kazakhstan	Aral	Cross-sectional	All breast cancers from 1999 to 2009	Breast cancer	Pollutions in Aral Lake can increase the number of breast cancers
Mamyrbayev A, et al: 2016 (25 )	Kazakhstan	Aral	case-control	All cancers from 2003 to 2014	All Cancers	Increase in cancers around Aral Lake can be due to Nickel and Cadmium (R= 0.8)
Herbst S, et al: 2008 (10 )	Uzbekistan	Aral	Cross-sectional	1282 citizens	Diarrhea	Unhealthy life situations, unhealthy water, domestic domains and other health issues were the main causes of diarrhea
Herbst S, et al: 2003 (26)	Uzbekistan	Aral	Cross-sectional	2239 samples of drinking water	diarrhea	Diarrhea was decreased despite many existing problems in quality of drinking water in rural areas
Kaneko K, et al: 2002 (27)	Uzbekistan	Aral	Cross-sectional	205 children	Kidney and urological diseases (hypercalciuria)	The prevalence of hypercalciuria around Aral lake was too high when compared with controls
Riabinskii VS, et al: 1993 (28)	Uzbekistan	Aral	case-control	178 patients	Kidney and urological diseases	By getting closer to the lake the prevalence of kidney diseases increased
Kaneko K, et al: 2003(29)	Uzbekistan	Aral	case-control	205 children	Kidney and urological diseases	The prevalence of kidney diseases around Aral lake was too high when compared with controls
Arustamov D, et al: 2001 (30 )	Uzbekistan	Aral	case-control	1817 rural people	Kidney and urological diseases	The prevalence of kidney diseases around Aral Lake was too high probably due to environmental and water pollutions
Love GJ, et al: 1982 (31 )	USA	Great Salt Lake	case-control	250 families	Respiratory problems	There was no linkage between Great Salt Lake drying and respiratory problems
Gomez SR, et al: 1992 (32 )	Canada	Old Wives Lake	case-control	300 participants	Respiratory problems	In regions affected by lake drying, winds contained alkaline soil causing respiratory problems
Kunii O, et al: 2003 (33 )	Uzbekistan	Aral	case-control	337 children	Respiratory problems	Respiratory problems in children around Aral Lake was more frequent than other regions
Bennion P, et al: 2007 (34 )	Uzbekistan	Aral	Cross-sectional	1499 children	Respiratory problems	Major respiratory problems including asthma had no significant relationship with exposure to Lake soil
Kislitskaya VN, et al: 2015 (35 )	Kazakhstan	Aral	Cross-sectional	-	Reproduction problems	Aral Lake pollution can have adverse effects on reproduction system
Turdybekova YG, et al: 2015 (36 )	Kazakhstan	Aral	Cross-sectional	1406 women	Reproduction problems	Aral Lake pollution can have adverse effects on women’s reproduction system including menstrual and menopausal disorders
Kislitskaya VN, et al: 2014 (37 )	Kazakhstan	Aral	Cross-sectional	251 men	Reproduction problems	Aral Lake pollution can have adverse effects on reproduction system
Kultanov BZ, et al: 2016 (12 )	Kazakhstan	Aral	Cross-sectional	1010 men	Reproduction problems	Aral Lake pollution can have adverse effects on reproduction system
Mazhitova Z, et al: 1998 (38)	Uzbekistan	Aral	Cross-sectional	12 hospitalized children	Other problems (growth and thyroid problems)	No relation was observed between chlorine resources and growth and thyroid problems
Crighton EJ, et al: 2003 (39 )	Uzbekistan	Aral	Cross-sectional	1118 participants aged above 18 years old	Other problems (psychological problems)	Environmental problems caused by Aral Lake drying can have psychological problems in addition to physical effects
Crighton EJ, et al: 2003 (40 )	Uzbekistan	Aral	Cross-sectional	881 participants aged above 18 years old	Other problems (self-assessment of whole health profile)	Residents around Aral Lake believed that they were not well in general

Out of 22 included articles, 17 studies in their conclusion pointed out that lake drying has adverse effects on human health. While 5 others were not able to demonstrate a relationship between human health and lakes drying.

**Fig. 2:**
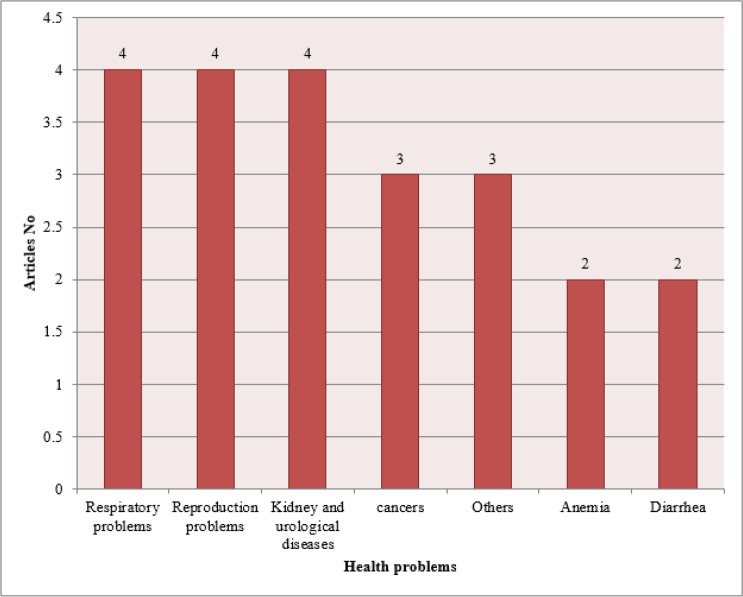
The most important health problems coming from lakes drying mentioned in studied papers

According to the effects of lakes drying, we classified the studies into 7 categories (Fig. 2). Having 4 studies for each category, the respiratory problem, reproductive system problems, kidney, and urological diseases ranked the highest.

### Anemia

Two articles were about anemia. Both were about Aral Lake drying in Kazakhstan. In a study, students’ blood were examine to measure HGB level and nutritional information were survey using 24-h nutritional reminder. Results showed iron level in consumed food was adequate (6.9% to 7.2%) while its absorption was below the normal level (3.1% to 4.4%). Blood samples of 46 students were taken and about 49% of students had anemia and 22% had iron deficiency. Moreover, 34% of students with iron deficiency had anemia (22, 23).

### Cancers

Three articles were related to cancers. All childhood cancers investigated and results show mortality rate around Aral Lake was higher than Kazalinsk (the control) (13). Mortalities of childhood cancers were about 1.3–2.7 per 100000 people. The most prevalent cancers were leukemia, skin cancer, gastrointestinal cancers and brain, and central nervous system cancers.

In a study on all breast cancers, the results showed a growth rate of breast cancers around Aral Lake (24). Mamyrbayev A and colleagues findings showed more prevalence of almost all types of cancers in studied areas including esophageal, gastric, lung, liver and breast cancers. Mortality rate of cancers in long term period around Lake Aral was 1.5 times higher than in other areas (25).

### Diarrhea

We found two articles about diarrhea. In 12-wk follow-up period in summer (96432 d/person) and in a 4-week follow-up in winter (32816 d/person) there were 593 cases of diarrhea in 313 participants out of 1148 total participants. Overall, 139 (44%) had two or more experiences (10). The average time of diarrhea for women in summer was 2.7 d and 2.9 for men. Children under 2 years old were more exposed to diarrhea. In a study, despite unhealthy water, the emergence rate of Diarrhea in 1999–2001 declined and insufficient reports were the main reason behind that (26).

### Kidney and Urological diseases

Four articles were about kidney diseases. Urine samples were taken to extract Sodium per creatinine, (uNA/Cr) and calcium per creatinine (uCa/Cr). Both items were higher in urine samples of children of Aral Lake. Calcium level higher than 0.703 was seen among 79 (38.6%) out of 205 participants, while it was seen only in 24 participants out of 187 (12.8) in control (27). Besides, organic acids and trace elements were measured in blood and urine samples of 178 kidney patients using chromato-mass spectrometry, absorption plasma spectrophotometry and ion-exchange chromatography. The number of lithogenic elements in urine and blood samples was categorized according to geographical location/distance from Aral Lake (including safe or far region, semi-safe or relatively near to the Lake and danger zone or close to the Lake). Then dangerous identified items according to the mentioned categorization. High or average level of danger was 26% in safe zone and was respectively 28.5% and 42.5% in semi-safe and danger zones (28). Kaneko and colleagues study results show both NAG and BMG were higher in Aral children when compared with the control (NAG: 0.77 (0.58) and 0.62 (0.37) U/mmol Cr; BMG: 41.8 (54.8) and 22.5 (20.4) mg/mmol Cr, respectively; mean (SD), p, 0.01). Children with abnormal NAG in Aral lake group were higher in percentage when compared with the control group (7.9% vs2.6%) (29). In Arustamov and colleagues (2001) study the prevalence rate of kidney and urological problems was estimated to be 0.7% (30).

### Respiratory problems

We found four articles on this issue. Love and colleagues examined the relation between sulfur dioxide air pollution with respiratory problems and showed that there was no linkage between pollution caused by Great Salt Lake drying and respiratory problems (31). Gomez and colleagues in Canada investigated the effect of winds containing alkaline soil on respiratory problems. The number of coughs, chest wheeze, chronic coughs; chronic chest wheeze, chronic eye problems and chronic nasal problems were higher in those exposed to alkaline winds (32). Another study about the adverse effects of Aral Lake drying on the respiratory showed that the prevalence of coughing and chronic chest wheezing was higher in the first group. The predicted Forced Vital Capacity (FVC %) was lower among in the first group (96.6% vs. 100.5%). Moreover, the predicted prevalence of lung diseases was higher in the first group (10.6% vs. 2.6%) (33). Chest wheezing was predicted to be 4.2%. No meaningful difference was seen between the amount of dust and respiratory problems. However, in summer there was a meaningful relationship between the amount of dust and predicted forced expiratory volume in 1s (34).

### Reproductive system problems

We found 4 articles in this regard. In a study the researchers calculated activity of adenosine deaminase (ADA) and activity of catalase (CAT) indexes by sampling the sperm of those men exposed to Aral Lake drying pollution at least for 5 years. ADA and CAT indexes were changed due to increase in oxidative stress and development of the pathological processes (35). About 39% of the participants had delayed/irregular periods. Menopause age was found to be decreased. About one-third of studied women had inflammation in their sexual organ. About 25% had experience of intra-abdominal embryo death or unsuccessful first pregnancy (36). In the age range of 40–45 there was an increase in MDA (malondialdehyde) level. Also with the increase in age, there was an increase in MMP (medium-molecular peptides) weight in men’s sperm.

There was also a change in lipid peroxidation process of their sperm (37). Moreover, 21% to 42% of total 1010 participants had transparent sperm (indicating low sperm count). Most of the participants had low sperm count in 1 ml of their sperm (<70 million). ASF (Acid-Souble Fractions) of these men was higher than the other parts residents (12).

### Other issues

This section deals with the studies not placed under the former categories. In a study with the aim of investigating the effect of chlorine pollutions by Aral Lake drying on growth indexes and thyroid function, they gathered the data and characteristics of 12 hospitalized children severely exposed to lake drying. PCBs (Polychlorinated Biphenyls) levels in these children were two to four times higher than the normal and healthy Swedish children. The average height of the exposed children also was lower. BMI of these children had a reverse relationship with the level of PCBs, DDT (Dichlorodiphenyltrichloroethane) and DDE (Dichlorophenyldichloroethylene) of their blood. Thyroxine and TSH level indicated no thyroid malfunction (38). Crighton and colleagues study with aim of investigating the effect of Aral Lake drying on their mental/psychological conditions show 41% of the participants had major concerns about the effects of Aral Lake drying on mental/psychological wellbeing. Moreover, 48% attributed their physical problems to their mental pressures and stress. The difference between men and women in this regard was indeed meaningful (39). In another study the authors tried to evaluate the general health profile of people residing around Aral Lake based on their own viewpoints. Utilizing different questionnaires they studied 881 participants health profile. Results showed that more than half of participants (55%) expressed their health condition to be weak or very bad. Only one percent said their health was very well. The main negative affecting factors on people’s attitudes toward their health were psychological problems, environmental concerns and age. Having a schema of health condition by a person himself/herself is a very strong predictor of mortalities and morbidities caused by diseases (40).

## Discussion

Finally, 22 articles included in the study of which 17 articles stated that lakes drying have adverse effects on human health.

Most studies discussed Aral Lake drying. It was one of the four biggest Lakes all around the world acquiring a total space of 68000 km/m^2^. However, in 1960s the Soviet Union government started to divert Amu Darya and SyrDarya rivers to Qara Qum desert for agricultural purposes. This resulted in the gradual shrinkage of the Lake. It was then divided into two smaller Southern Aral and Northern Aral Lakes in 1987. In 2004 the Lake reached to its 25% of space and its salinity became 5 times higher. The two lakes then went into four smaller lakes in 2007. These four lakes compromised 10% of the main Lake. In 2009 satellite images showed that two of the lakes have been totally dried and the others get much smaller. Ecosystem pollution was the main challenge of this area. Consequent to drying, Aral Lake area inflicted with tremendous amount of pollution that resulted in the outbreak of diseases. Today, Aral Lake drying has been regarded as one of the major global ecosystem disasters (6, 40, 41).

Children were more focused by researchers among target groups. The main reason was their vulnerability against adverse effects of lakes drying and natural disaster in general. Children were more accessible by being present at schools and for this probable reason researchers were tended to have more focus on them. Despite such concentration on children, there are not any clear and strong evidence about the adverse effects of lakes drying on children’s health. In this respect, anemia, diarrhea and exposure to toxic materials were the main challenges when talking about children’s health and its linkage to Aral Lake drying (42). However, there is no strong evidence to confirm the hypothesis and further comprehensive studies are needed (43).

In the present review, the majority of studies (17 out of 22) explicitly confirmed the adverse effects of Lake drying on human health. The present study only investigated the direct effects of lakes drying on humans’ health. Certainly, further to direct consequences, lakes drying have indirect effects on health. Of these indirect effects, one can mention the economic, psychological and long term indirect effects on human conditions (44). In the present review also some studies excluded measured the level of chemical and non-chemical substances by lake drying were transferred into human body through wind, water, and agricultural products because they did not assess any relationship between the substances and humans’ health (45–49). Of course, that is another area for lake drying to affect human health and thus needs to be investigated.

We classified studies based on different effects of lakes drying into 7 categories including respiratory problems, reproductive system disorders, kidney and urological diseases, cancers, anemia, diarrhea and other problems. Despite investigations in each field, it seems there is not yet any firm and clear evidence to either accept or reject the adverse effect of lake drying on human health and some further studies should be carried out. Throughout the present study, some other important problems caused by lake drying have remained neglected that are: eye diseases, cardiovascular diseases, neurological diseases and ENT diseases.

Almost all studies were cross-sectional and case-control. Despite the notable stands of these types of studies in epidemiological issues, they have not enough level of evidence and practicality to demonstrate the causal relationship between risk factor and diseases emergence. None of the mentioned studies did use appropriate statistical indexes such as odds ratio or attributable risk. One of the major epidemiological studies that have high efficiency in demonstrating the causal relation between risk factor and diseases are cohort or prospective studies. Prospective cohort studies are much focused when there is an intention to approach real findings in holistic observation (50–53). Therefore, researchers and policymakers should design and undertake well-structured mid-term and long-term cohort studies instead of cross-sectional research with small sample size in limited areas that acquire low level of evidence and efficiency.

The present study in fact faced with some limitations. The most important one was that almost studies only focused on Aral Lake drying. This issue, we think, can affect the generalizability of findings.

## Conclusion

Explained by most studies, lakes drying could have adverse effects on humans’ health but since they were cross-sectional and retrospective they had low level of evidence and since they were limited to specific geographical location they were not successful to provide us with strong and clear evidence to either accept or reject with high certainty the adverse effects of lake drying on human’s health. In order to have firm evidence we suggest that researchers and policymakers should design and undertake well-structured mid-term and long-term cohort studies instead of cross-sectional research with small sample size in limited areas that have low level of evidence and efficiency.

## Ethical considerations

Ethical issues (Including plagiarism, informed consent, misconduct, data fabrication and/or falsification, double publication and/or submission, redundancy, etc.) have been completely observed by the authors.

## References

[1] Ruijten M (2007). The Dutch experience with Health Impact Assessment of disasters. Eur J Public Health, 17(1):5–6. 17005510 10.1093/eurpub/ckl079

[2] Verger P Ruijten M Russell D (2007). Better planning for health impact assessment of disasters. Eur J Public Health, 17(1):3. 10.1093/eurpub/ckl076 17068004

[3] Field C Barros V Stocker T Qin D Dokken D Ebi K (2012). Managing the Risks of Extreme Events and Disasters to Advance Climate Change Adaptation. A Special Report of Working Groups I and II of the Intergovernmental Panel on Climate Change. Cambridge University Press, Cambridge, UK, and New York, NY, USA.

[4] Jackson SF Fazal N Gravel G Papowitz H (2017). Evidence for the value of health promotion interventions in natural disaster management. Health Promot Int, 32(6): 1057–1066. 27099239 10.1093/heapro/daw029

[5] Stanley SE Faulkenberry JB (2014). Force health protection support following a natural disaster: the 227th Medical Detachment’s role in response to Superstorm Sandy. J Spec Oper Med, 14(4):106–112. 25399378 10.55460/LTRU-9D2L

[6] Micklin P (2007). The Aral Sea Disaster. Annu Rev Earth Planet Sci, 35:47–72.

[7] Eimanifar A Mohebbi F (2007). Urmia Lake (Northwest Iran): a brief review. Saline Systems, 3:5. 17506897 10.1186/1746-1448-3-5 PMC1884160

[8] Gholampour A Nabizadeh R Hassanvand MS (2015). Characterization of saline dust emission resulted from Urmia Lake drying. J Environ Health Sci Eng, 13:82. 26617986 10.1186/s40201-015-0238-3 PMC4663037

[9] Cox HS Kubica T Doshetov D (2005). The Beijing genotype and drug resistant tuberculosis in the Aral Sea region of Central Asia. Respir Res, 6:134. 16277659 10.1186/1465-9921-6-134 PMC1299328

[10] Herbst S Fayzieva D Kistemann T (2008). Risk factor analysis of diarrhoeal diseases in the Aral Sea area (Khorezm, Uzbekistan). Int J Environ Health Res, 18(5):305–321. 18821371 10.1080/09603120701834507

[11] Izhitskiy AS Zavialov PO Sapozhnikov PV (2016). Present state of the Aral Sea: diverging physical and biological characteristics of the residual basins. Sci Rep, 6:23906. 27032513 10.1038/srep23906 PMC4817148

[12] Kultanov BZ Dosmagambetova RS Ivasenko SA (2016). The Study of Cellular and Molecular Physiological Characteristics of Sperm in Men Living in the Aral Sea Region. Open Access Maced J Med Sci, 4(1):5–8. 27275320 10.3889/oamjms.2016.007 PMC4884251

[13] Mamyrbayev A Dyussembayeva N Ibrayeva L (2016). Features of Malignancy Prevalence among Children in the Aral Sea Region. Asian Pac J Cancer Prev, 17(12):5217–5221. 28125864 10.22034/APJCP.2016.17.12.5217 PMC5454661

[14] Mischke S Liu C Zhang J (2017). The world’s earliest Aral-Sea type disaster: the decline of the Loulan Kingdom in the Tarim Basin. Sci Rep, 7:43102. 28240223 10.1038/srep43102 PMC5327390

[15] Sakiev KZ (2014). [[On evaluation of public health state in Aral Sea area]. Med Tr Prom Ekol, (8):1–4. 25549450

[16] Ono DM Hardebeck E Parker J Cox BG (2000). Systematic biases in measured PM10 values with U.S. Environmental Protection Agency-approved samplers at Owens Lake, California. J Air Waste Manag Assoc, 50(7):1144–1156. 10939208 10.1080/10473289.2000.10464163

[17] Pikuta EV Itoh T Krader P (2006). Anaerovirgula multivorans gen. nov., sp. nov., a novel spore-forming, alkaliphilic anaerobe isolated from Owens Lake, California, USA. Int J Syst Evol Microbiol, 56(Pt 11):2623–2629. 17082402 10.1099/ijs.0.64198-0

[18] S Khan K Kunz R Kleijnen J Antes G (2011). Systematic reviews to support evidence-based medicine. edn, vol. 2.

[19] Poorolajal J Tajik P Yazdizadeh B (2009). Quality Assessment of the Reporting of Cohort Studies before STROBE Statement. Iran J Epidemilogy, 5(1):17–26.

[20] von Elm E Altman DG Egger M (2007). The Strengthening the Reporting of Observational Studies in Epidemiology (STROBE) statement: guidelines for reporting observational studies. Ann Intern Med, 147(8):573–577. 17938396 10.7326/0003-4819-147-8-200710160-00010

[21] von Elm E Altman DG Egger M (2007). The Strengthening the Reporting of Observational Studies in Epidemiology (STROBE) statement: guidelines for reporting observational studies. PLoS Med, 4(10):e296. 17941714 10.1371/journal.pmed.0040296 PMC2020495

[22] Hashizume M Kunii O Sasaki S (2003). Anemia and iron deficiency among schoolchildren in the Aral Sea region, Kazakhstan. J Trop Pediatr, 49(3):172–177. 12848209 10.1093/tropej/49.3.172

[23] Hashizume M Shimoda T Sasaki S (2004). Anaemia in relation to low bioavailability of dietary iron among school-aged children in the Aral Sea region, Kazakhstan. Int J Food Sci Nutr, 55(1):37–43. 14630590 10.1080/09637480310001642466

[24] Mamyrbayev A Djarkenov T Dosbayev A (2016). The Incidence of Malignant Tumors in Environmentally Disadvantaged Regions of Kazakhstan. Asian Pac J Cancer Prev, 17(12):5203–5209. 28125862 10.22034/APJCP.2016.17.12.5203 PMC5454659

[25] Bilyalova Z Igissinov N Moore M (2012). Epidemiological evaluation of breast cancer in ecological areas of Kazakhstan--association with pollution emissions. Asian Pac J Cancer Prev, 13(5):2341–2344. 22901219 10.7314/apjcp.2012.13.5.2341

[26] Herbst S Kistemann T Fayzieva D (2003). Comparison of the incidence of diarrhoeal diseases in two regions of the Aral Sea area, Uzbekistan. In: Environmental health risk II, Brebbia C Fayzieva D (eds), pp 153–161. Southampton: WIT Press.

[27] Kaneko K Chiba M Hashizume M (2002). Extremely high prevalence of hypercalciuria in children living in the Aral Sea region. Acta Paediatr, 91(10):1116–1120. 12434899 10.1080/080352502760311638

[28] Riabinskii VS Doschanov E Istratov VG (1993). [Risk factors for the occurrence of nephrolithiasis in the Aral Sea region]. Urol Nefrol (Mosk), (4):19–21. 8310558

[29] Kaneko K Chiba M Hashizume M (2003). Renal tubular dysfunction in children living in the Aral Sea Region. Arch Dis Child, 88(11):966–968. 14612357 10.1136/adc.88.11.966 PMC1719339

[30] Arustamov D Fayzieva D Nurullaev R Klyopov Y (2001). Study on the rate of the urolithiasis in the Aral Sea area and the quality of potable water pp In: Environmental health risk, Fajzieva D Brebbia C (eds), pp 105–111. Southampton: WIT Press.

[31] Love GJ Lan SP Shy CM (1982). A study of acute respiratory disease in families exposed to different levels of Air pollution in the Great Salt Lake basin, Utah, 1971–1972 and 1972–1973. Environ Health Perspect, 44:165–174. 7084150 10.1289/ehp.8244165 PMC1568968

[32] Gomez SR Parker RA Dosman JA McDuffie HH (1992). Respiratory health effects of alkali dust in residents near desiccated Old Wives Lake. Arch Environ Health, 47(5):364–369. 1444599 10.1080/00039896.1992.9938376

[33] Kunii O Hashizume M Chiba M (2003). Respiratory symptoms and pulmonary function among school-age children in the Aral Sea region. Arch Environ Health, 58(11):676–682. 15702891 10.3200/AEOH.58.11.676-682

[34] Bennion P Hubbard R O’Hara S (2007). The impact of airborne dust on respiratory health in children living in the Aral Sea region. Int J Epidemiol, 36(5):1103–1110. 17911152 10.1093/ije/dym195

[35] Kislitskaya VN Kenzhin ZD Kultanov BZ (2015). Disturbance of Antioxidant Enzymes and Purine Metabolism in the Ejaculate of Men Living in Disadvantaged Areas of Kyzylorda Region. Open Access Maced J Med Sci, 3(3): 489–492. 27275276 10.3889/oamjms.2015.085 PMC4877845

[36] Turdybekova YG Dosmagambetova RS Zhanabayeva SU (2015). The Health Status of the Reproductive System in Women Living In the Aral Sea Region. Open Access Maced J Med Sci, 3(3):474–477. 27275273 10.3889/oamjms.2015.078 PMC4877842

[37] Kislitskaya VN Yesilbayeva BT Kubayev AB (2014). The Level of Molecules of Average Mass and Lipid Peroxidation Processes in the Semen of Men of Reproductive Age in Kazalinsk District of Aral Sea Region. World J Med Sci, 11(4):552–554.

[38] Mazhitova Z Jensen S Ritzen M Zetterstrom R (1998). Chlorinated contaminants, growth and thyroid function in schoolchildren from the Aral Sea region in Kazakhstan. Acta Paediatr, 87(9):991–995. 9764896 10.1080/080352598750031671

[39] Crighton EJ Elliott SJ Meer J Small I Upshur R (2003). Impacts of an environmental disaster on psychosocial health and well-being in Karakalpakstan. Soc Sci Med, 56(3):551–567. 12570973 10.1016/s0277-9536(02)00054-0

[40] Sousa A Hill K Dal Poz MR (2010). Sub-national assessment of inequality trends in neonatal and child mortality in Brazil. Int J Equity Health, 9:21. 20815875 10.1186/1475-9276-9-21 PMC2944212

[41] Dickson KE Simen-Kapeu A Kinney MV (2014). Every Newborn: health-systems bottlenecks and strategies to accelerate scale-up in countries. Lancet, 384(9941):438–454. 24853600 10.1016/S0140-6736(14)60582-1

[42] Crighton EJ Elliott SJ Upshur R van der Meer J Small I (2003). The Aral Sea disaster and self-rated health. Health Place, 9(2):73–82. 12753790 10.1016/s1353-8292(02)00017-5

[43] Crighton EJ Barwin L Small I Upshur R (2011). What have we learned? A review of the literature on children’s health and the environment in the Aral Sea area. Int J Public Health, 56(2):125–138. 20976516 10.1007/s00038-010-0201-0 PMC3066395

[44] Beard J (2006). DDT and human health. Sci Total Environ, 355(1–3):78–89. 15894351 10.1016/j.scitotenv.2005.02.022

[45] Hooper K Hopper K Petreas MX (1997). Analysis of breast milk to assess exposure to chlorinated contaminants in Kazakstan: PCBs and organochlorine pesticides in southern Kazakstan. Environ Health Perspect, 105(11):1250–1254. 9370517 10.1289/ehp.971051250 PMC1470329

[46] Muntean N Jermini M Small I (2003). Assessment of dietary exposure to some persistent organic pollutants in the Republic of Karakalpakstan of Uzbekistan. Environ Health Perspect, 111(10):1306–11. 12896851 10.1289/ehp.5907 PMC1241611

[47] Jensen S Mazhitova Z Zetterstrom R (1997). Environmental pollution and child health in the Aral Sea region in Kazakhstan. Sci Total Environ, 206(2–3):187–193. 9394482

[48] O’Hara SL Wiggs GF Mamedov B Davidson G Hubbard RB (2000). Exposure to airborne dust contaminated with pesticide in the Aral Sea region. Lancet, 355(9204):627–628. 10696990 10.1016/S0140-6736(99)04753-4

[49] Ataniyazova OA Baumann RA Liem AK (2001). Levels of certain metals, organochlorine pesticides and dioxins in cord blood, maternal blood, human milk and some commonly used nutrients in the surroundings of the Aral Sea (Karakalpakstan, Republic of Uzbekistan). Acta Paediatr, 90(7):801–808. 11519985

[50] Kader M Perera NK (2014). Socio-economic and nutritional determinants of low birth weight in India. N Am J Med Sci, 6(7):302–308. 25077077 10.4103/1947-2714.136902 PMC4114006

[51] Monaco J (2013). Cohort Study. In: Gellman MD Turner JR Encyclopedia of Behavioral Medicine, edn. New York, NY: Springer New York. Pp: 454–455.

[52] Liddell FD (1988). The development of cohort studies in epidemiology: a review. J Clin Epidemiol, 41(12):1217–37. 3062142 10.1016/0895-4356(88)90027-3

[53] Checkoway H Pearce N Dement JM (1989). Design and conduct of occupational epidemiology studies: II. Analysis of cohort data. Am J Ind Med, 15(4):375–94. 2658566 10.1002/ajim.4700150403

